# Site-Specific Trends in Tumor Distribution, Treatment Patterns, and Survival in Head and Neck Cancer: A SEER Database Analysis *

**DOI:** 10.1055/a-2884-1665

**Published:** 2026-10-14

**Authors:** Christopher Stewart, JB Eyring, Abbigail Niewchas, Joseph Hanny, Grant Sanders, Alex Otto, Randall Hansen, Kent McIntire

**Affiliations:** 1 Department of MedicineKansas City UniversityJoplinMOUnited States; 2 Department of MedicineUniversity of UtahSalt Lake CityUTUnited States; 3 Department of OtolaryngologyFreeman Health SystemJoplinMOUnited States

**Keywords:** head and neck surgery, head and neck cancer, combination treatment

## Abstract

**Introduction:**

Head and neck cancer (HNC) management has evolved significantly with the introduction of organ-preservation protocols and changing disease etiology.

**Objective:**

To analyze site-specific trends in tumor distribution, staging, treatment modality, and survival outcomes using the Surveillance, Epidemiology, and End Results (SEER) program.

**Methods:**

A retrospective analysis of 288,132 malignant HNC cases from the SEER program (2000–2021) was performed. Survival analyses were restricted to cases diagnosed between 2000 and 2015 to allow for adequate follow-up. The anatomical sites included the salivary gland, larynx, the oral cavity, the lip, the tongue, the nasopharynx, the oropharynx, and the hypopharynx. Trends in tumor proportion, clinical stage, and treatment modality were assessed using linear regression. Five-year survival trends were analyzed using multivariate logistic regression.

**Results:**

The proportion of tongue-cancer diagnoses increased by 12.2% ( *p* < 0.001), while that of laryngeal cancer decreased by 10.0% ( *p* < 0.001). Treatment patterns shifted significantly, with the rates of surgery combined with radiation (−11.0%; *p* < 0.001) and radiation alone (−7.6%; *p* < 0.001) declining, while that of chemoradiation increased (+16.9%; *p* < 0.001). The 5-year survival rates improved for hypopharynx (8.5%; *p* < 0.001), tongue (8.5%; *p* < 0.001), and oropharynx (8.0%; *p* < 0.01) cancers.

**Conclusion:**

The management of HNC has shifted toward multimodal therapies involving chemotherapy, coinciding with improved survival in pharyngeal and oral subsites. The rising proportion of tongue and oropharyngeal cases may reflect changes in underlying etiologic factors, including human papillomavirus (HPV)-associated disease, although HPV-specific effects cannot be directly assessed in the SEER program.

## Introduction

Head and neck cancers (HNCs) include malignancies of the pharynx, larynx, oral cavity, and associated glands. In 2025, an estimated 20,040 new cases of tongue, 15,730 of mouth, 21,640 of pharyngeal, 13,020 of laryngeal, and 2,250 of other-oral-cavity cancer are expected in the United States (US). 1 Primary tumor site, histologic subtype, and the Tumor, Node, Metastasis (TNM) classification remain major determinants of treatment and outcomes. 2 Advances in molecular diagnostics and the identification of the human papillomavirus (HPV) as a key etiological factor have reshaped prognostic models and treatment guidelines. 3

Population-based trend analyses demonstrate that survival differs by subsite: the rate of 5-year survival rose from 39 to 73% for tongue cancer (1975–2017), but it declined for laryngeal cancer (from 66 to 62%) over a similar period. 1 Such variability underscores the need for continued subsite-specific research. To better understand how tumor site, histology, stage, and etiological factors shape subsite-specific incidence and outcomes in HNC, researchers have relied on detailed epidemiologic datasets to assess trends over time. The Surveillance, Epidemiology, and End Results (SEER) program provides population-base data enabling detailed trend analyses. Though previous site-specific analyses have established more precise incidence and prognosis trends than generalized HNC studies, data from the turn of the century is yet to be reported on. 4 Novel targeted and immunotherapies approved in the last 20 years, as well as rising prevalence of newly-discovered etiologies of HNC cancers, prompt further investigation into possible alterations in management and outcomes. 3 5 The current study examines site-specific tumor distribution and treatment patterns for HNCs from 2000 to 2021, and site-specific survival trends from 2000 to 2015.

## Methods

Data were obtained from the SEER program in September 2024 using their 17 registries, which cover approximately 26% of the US population.

### Inclusion Criteria

Tumors were classified using codes from the International Classification of Diseases, Tenth Revision (ICD-10; C00–C14, C30–C32) and grouped into 9 anatomical categories (Appendix 1). Eligible cases included malignant tumors of the lip, tongue, oral cavity, salivary glands, oropharynx, nasopharynx, hypopharynx, larynx, and other oral sites diagnosed between 2000 and 2021. Survival analyses were restricted to cases diagnosed between 2000 and 2015 to ensure adequate 5-year follow-up. No restrictions were placed on age. Gender was restricted to male and female.

### Variables

Data from cases meeting the inclusion criteria was first analyzed by demographic information, including gender, age, year of diagnosis, tumor site, clinical stage at presentation, and treatment modality. Gender was categorized as male or female. Age was grouped as < 45, 45–54, 55–64, 65–74, and ≥ 75 years. The year of diagnosis was any single year between 2000 and 2021. The clinical stages at presentation were categorized as *localized* , *regional* , or *distant* based on the SEER Summary Staging Guide, 6 while those lacking information on staging were categorized as *unstaged* . The tumor sites analyzed include the lip, tongue, gums, mouth, palate, parotid gland, other major salivary glands, tonsils, oropharynx, nasopharynx, hypopharynx, piriform sinus, nasal cavity, middle ears, accessory sinuses, and the larynx. The treatments included surgery only; radiation only; surgery and radiation; chemotherapy and radiation; or surgery, chemotherapy, radiation, or ‘other’, which included cases in which chemotherapy alone was used for treatment, or the treatment information was not available in the database. Treatments were grouped as: *surgery only* , *radiation only* , *surgery + radiation* , *chemoradiation only* , *surgery + chemoradiation* , or *other/unknown* .

### Statistical Analysis

Data was exported from SEER (in XLSX file format) and analyzed in SAS OnDemand for Academics (SAS Institute Inc.) software, version 9.4. The proportional distribution of tumor sites was calculated as a percentage of the total HNC cohort for each year. All tumor-site trends were reported as proportions of the overall HNC cohort, not as incidence rates. Temporal trends in the proportion of diagnoses, stage at presentation, and treatment modality were assessed using linear regression. Chi-square tests were used to evaluate associations involving categorical variables.

The 5-year cancer-specific survival was evaluated using multivariate logistic regression, with survival status (alive versus cancer-specific death at 5 years) as the dependent variable. The year of diagnosis was included as the primary independent variable of interest. The model was adjusted for age (categorical), gender, stage at diagnosis, and treatment modality to account for potential confounding. Adjustment for stage at diagnosis was performed to assess whether the observed survival trends were independent of potential stage migration over time.

To evaluate temporal trends in survival at the population level, yearly 5-year cancer-specific survival rates were calculated and analyzed using linear regression, with year of diagnosis as the independent variable. Cases with unknown cause of death and those diagnosed after 2015 were excluded to ensure complete 5-year follow-up. Statistical significance was defined as *p* < 0.05.

Missing stage was categorized as *unstaged* , and treatment classified as *other* included chemotherapy-only and incomplete or missing treatment data; multivariate analyses were performed using complete-case data.

## Results

### Study Cohort and Baseline Characteristics

A total of 288,132 HNC patients met inclusion criteria from the SEER 17 registries ( Table 1 ). The cohort was predominantly male (71.6%), with most patients diagnosed after age 55. Case volume increased steadily over time, with the largest proportion of diagnoses occurring between 2015 and 2020. Tongue (22.3%), laryngeal (20.7%), and oropharyngeal (16.2%) cancers represented the most common tumor sites. At diagnosis, the highest proportion of tumors were classified as regional-stage disease (43.4%), followed by localized (36.3%) and distant disease (14.2%). Surgery alone (24.0%) and chemoradiation (23.4%) were the most frequently used treatment modalities. Key trends in the proportions of tumor distribution, stage at diagnosis, treatment, and survival are summarized in Table 2 .

**Table 1:** Distribution of cases according to the clinical demographic variables

Variable category	N	Percentage
**Gender**		
Male	206,370	71.62%
Female	81,762	28.38%
**Age at diagnosis (years)**		
< 45	20,126	6.98%
45–54	47,807	16.59%
55–64	83,674	29.04%
65–74	74,413	25.83%
≥ 75	62,112	21.56%
**Interval year**		
2000–2002	32,812	11.39%
2003–2005	33,753	11.71%
2006–2008	36,255	12.58%
2009–2011	38,862	13.49%
2012–2014	41,670	14.46%
2015–2017	44,003	15.27%
2018–2020	45,246	15.70%
2021	15,531	5.39%
**Tumor site**		
Tongue	64,353	22.33%
Larynx	59,611	20.69%
Oropharynx	46,764	16.23%
Other mouth	33,333	11.57%
Nasopharynx	24,984	8.67%
Salivary gland	24,029	8.34%
Lip	13,422	4.66%
Hypopharynx	11,525	4.00%
Oral cavity	10,111	3.51%
**Seer staging**		
Localized	104,514	36.27%
Regional	124,968	43.37%
Distant	40,979	14.22%
Unstaged	17,666	6.13%
Other/Unknown	5	0.00%
**Treatment**		
Surgery	69,021	23.95%
Chemotherapy + radiation	67,316	23.36%
Surgery + radiation	44,405	15.41%
Radiation	33,737	11.71%
Surgery + chemotherapy + radiation	28,291	9.82%
Chemotherapy	5,548	1.92%
Other	39,814	13.82%

**Table 2:** Summary of temporal trends by tumor site (2000–2021)

Tumor Site	Proportional representation of HNC trend	Stage-distribution trend	Treatment trend	5-year survival trend*
Lip	↓	↓ Localized; ↑ regional	↓ Surgery alone; ↑ surgery + RT and trimodal	No change
Oral cavity	↓	↑ Distant	↓ Surgery and RT; ↑ trimodal	↑
Tongue	↑	↑ Localized and regional	↑ Chemoradiation; ↓ surgery + RT	↑
Salivary gland	Modest but significant ↑	Stable	↓ Surgery and surgery + RT; ↑ trimodal	Small but significant ↑
Nasopharynx	↓	↑ Distant	↓ Surgery and RT; ↑ chemoradiation	No change
Hypopharynx	↓	Stable	↓ RT and surgery + RT; ↑ chemoradiation	↑
Larynx	↓	↑ Distant	↓ RT and surgery + RT; ↑ chemoradiation	Small but significant ↑
Oropharynx	↑	↑ Regional	↓ Surgery + RT; ↑ chemoradiation	↑
Other mouth/Pharynx	↑	↑ Regional and distant	↓ Surgery and RT; ↑ trimodal	Modest but significant ↑

**Abbreviations:** HNC, head and neck cancer; RT, radiation therapy.

**Notes:** *Survival trends based on diagnoses from 2000 to 2015. ↑ and ↓ indicate statistically significant directional trends over the study period based on linear regression ( *p* < 0.05). ‘No change’ indicates no significant temporal trend. ‘Modest’ or ‘minimal’ indicates a statistically significant trend of small absolute magnitude. ‘Trimodal’ indicates that the patient received surgery, chemotherapy, and radiation.

### Temporal Distribution of Tumor Sites

Between 2000 and 2021, substantial site-specific changes were observed in the proportional distribution of HNCs ( Fig. 1 , Table 2 ). Fig. 1 demonstrates a shift in proportional representation, with tongue and oropharyngeal cancers comprising a larger share of cases over time, while laryngeal and hypopharyngeal cancers comprised a smaller share. Other subsites demonstrated more modest or variable changes over time.

**Fig. 1:**
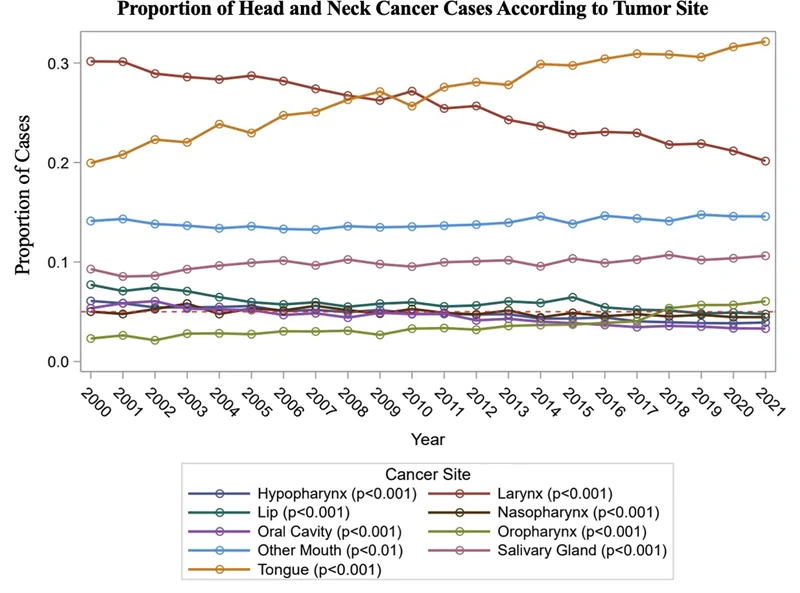
Proportion of cases of head and neck cancer according to tumor site by year of diagnosis from 2000–2021. The values represent the percentage of all cases of head and neck cancer diagnosed in each year attributable to each tumor site. Temporal trends were assessed using linear regression.

### Stage at Presentation

Trends in clinical stage at diagnosis varied by tumor site ( Tables 2 , 3 , Fig. 2 ), with Fig. 2 demonstrating a shift from localized toward regional-stage disease, accompanied by a modest increase in distant-stage presentation. Several subsites demonstrated increasing proportions of advanced-stage disease over time. Laryngeal cancers showed a significant decline in localized disease, with a corresponding increase in distant-stage presentation. Nasopharyngeal and other mouth/pharyngeal cancers exhibited marked increases in distant-stage disease, while oral cavity cancers demonstrated a smaller but statistically significant rise in distant-stage presentation.

**Table 3:** Trends in clinical stage (by the SEER staging system) according to the year of diagnosis

Rate of cases among HNC cases by year of diagnosis									
Region	Tumor-site group	2000–2002	2003–2005	2006–2008	2009–2011	2012–2014	2015–2017	2018–2020	*p* -value
Lip	Localized	88.72%	87.69%	86.51%	87.32%	86.04%	80.25%	64.07%	< 0.001 (-)
	Regional	3.56%	3.71%	5.40%	4.23%	4.62%	5.44%	12.21%	< 0.001 (+)
	Distant	1.48%	1.63%	1.50%	1.47%	1.05%	0.92%	1.38%	NS
	Data not available	6.23%	6.97%	6.59%	6.99%	8.29%	13.39%	22.35%	< 0.001 (+)
Hypopharynx	Localized	13.87%	14.87%	19.40%	17.30%	17.21%	16.30%	15.31%	NS
	Regional	64.84%	63.91%	60.37%	49.41%	40.01%	48.50%	62.41%	NS
	Distant	17.40%	16.37%	16.02%	30.29%	39.82%	29.98%	18.34%	NS
	Data not available	3.89%	4.85%	4.21%	3.00%	2.95%	5.22%	3.93%	NS
Larynx	Localized	60.21%	56.91%	56.76%	55.59%	54.57%	51.45%	50.66%	< 0.001 (-)
	Regional	28.07%	22.55%	19.77%	21.70%	22.29%	26.60%	27.05%	NS
	Distant	6.67%	13.22%	17.93%	18.02%	18.59%	16.54%	16.30%	< 0.01 (+)
	Data not available	5.04%	7.33%	5.54%	4.69%	4.55%	5.41%	5.99%	NS
Nasopharynx	Localized	1.69%	2.08%	1.81%	1.71%	1.68%	1.86%	1.52%	NS
	Regional	13.01%	10.74%	9.09%	8.78%	8.95%	10.60%	12.66%	< 0.01 (-)
	Distant	1.76%	4.89%	7.58%	7.93%	8.50%	7.10%	5.98%	< 0.001 (+)
	Data not available	1.09%	1.47%	1.59%	1.27%	1.30%	1.74%	1.65%	NS
Oral cavity	Localized	8.31%	7.36%	6.73%	8.10%	7.00%	6.58%	5.80%	NS
	Regional	9.16%	8.45%	8.09%	7.80%	7.37%	6.85%	7.74%	NS
	Distant	0.66%	0.93%	0.99%	1.45%	1.41%	1.26%	1.03%	< 0.01 (+)
	Data not available	0.86%	1.06%	0.78%	0.61%	0.58%	0.87%	1.10%	NS
Oropharynx	Localized	1.03%	1.40%	1.71%	1.81%	1.90%	2.14%	2.29%	< 0.01 (+)
	Regional	4.99%	5.87%	6.17%	6.98%	8.68%	10.68%	18.72%	p < 0.05 (+)
	Distant	1.61%	2.25%	2.79%	2.86%	3.80%	3.77%	4.42%	NS
	Data not available	0.66%	0.95%	0.93%	1.04%	0.95%	1.60%	1.90%	< 0.01 (+)
Other mouth	Localized	16.84%	17.31%	18.05%	19.42%	20.62%	22.09%	22.61%	NS
	Regional	22.97%	21.00%	21.23%	23.42%	26.24%	26.11%	24.30%	< 0.001 (+)
	Distant	2.89%	3.51%	4.63%	4.42%	5.44%	8.40%	12.56%	< 0.001 (+)
	Data not available	5.80%	6.26%	5.41%	5.25%	6.23%	6.57%	7.72%	0.05 (+)
Salivary gland	Localized	14.33%	15.02%	16.39%	17.35%	19.15%	21.53%	23.25%	NS
	Regional	10.30%	11.35%	13.18%	11.39%	12.25%	13.86%	16.05%	NS
	Distant	3.77%	5.34%	5.82%	7.13%	8.07%	7.14%	6.34%	NS
	Data not available	1.97%	2.98%	2.50%	2.72%	2.76%	3.25%	3.67%	NS
Tongue	Localized	27.18%	28.18%	32.66%	35.84%	38.62%	43.29%	43.00%	< 0.001 (+)
	Regional	33.57%	36.98%	43.46%	48.64%	56.20%	67.73%	80.93%	< 0.001 (+)
	Distant	5.80%	10.25%	12.75%	13.95%	18.17%	16.75%	13.96%	NS
	Data not available	4.04%	4.66%	3.94%	4.20%	4.60%	5.94%	6.84%	< 0.05 (+)

**Abbreviations:** HNC, head and neck cancer; NS, not significant; SEER, Surveillance, Epidemiology, and End Results program.

**Note:** *Percentages represent the proportion of all HNC cases diagnosed at each stage by tumor site, not stage distribution within each tumor site.

**Fig. 2:**
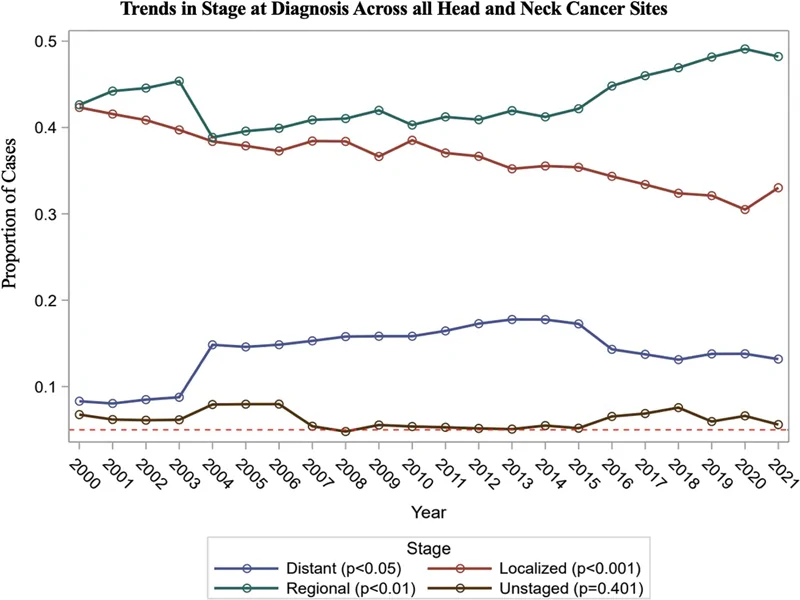
Trends in stage at diagnosis among all cases of head and neck cancer by year of diagnosis from 2000–2021. The values represent the proportion of all cases of head and neck cancer diagnosed at each SEER summary stage (localized, regional, distant, or unstaged) for each year.

In contrast, hypopharyngeal and salivary-gland cancers showed no significant change in stage distribution across the study period. Tongue cancers demonstrated a redistribution toward localized and regional-stage disease, without a significant increase in the proportion of distant-stage presentation. The proportion of unstaged cases increased modestly for select subsites, including the lip, tongue, oropharynx, and other mouth/pharynx cancers.

### Treatment Trends

Significant shifts in treatment patterns were observed across nearly all tumor sites between 2000 and 2021 ( Tables 2 , 4 , Fig. 3 ), with Fig. 3 illustrating increasing reliance on chemoradiation over time alongside declines in radiation alone and surgery-radiation combinations. Trimodal therapy increased for select subsites, and surgery alone decreased modestly across most tumor sites. The proportion of cases categorized as *other* treatment (chemotherapy alone or missing data) increased significantly across all tumor sites, resulting in a growing proportion of cases with incomplete treatment classification over time that may influence the interpretation of temporal treatment trends.

**Table 4:** Trends in treatment approach according to the tumor site and year of diagnosis

Rate of cases among HNC cases by year of diagnosis									
Region	Treatment	2000–2002	2003–2005	2006–2008	2009–2011	2012–2014	2015–2017	2018–2020	*p* -value
**Lip**	Surgery	84.97%	83.81%	82.91%	78.41%	76.54%	74.09%	66.90%	< 0.001 (-)
	Radiation	2.82%	3.04%	2.04%	3.33%	2.78%	2.26%	2.23%	NS
	Chemotherapy + radiation	0.25%	0.11%	0.42%	0.39%	0.21%	0.31%	0.29%	NS
	Surgery + radiation	5.79%	5.68%	5.04%	5.02%	5.35%	6.36%	7.67%	< 0.001 (+)
	Surgery + chemotherapy + radiation	0.45%	0.45%	1.32%	1.07%	0.89%	1.54%	1.55%	< 0.05 (+)
	Other/Unknown	5.74%	6.91%	8.27%	11.78%	14.23%	15.44%	21.36%	< 0.001 (+)
**Hypopharynx**	Surgery	7.85%	5.35%	5.04%	7.06%	7.22%	6.37%	6.21%	NS
	Radiation	19.71%	15.87%	13.53%	11.62%	12.81%	14.64%	12.83%	< 0.001 (-)
	Chemotherapy + radiation	28.59%	42.07%	45.95%	47.78%	46.04%	43.60%	43.86%	< 0.05 (+)
	Surgery + radiation	18.19%	9.40%	4.47%	4.06%	4.15%	4.71%	3.38%	< 0.001 (-)
	Surgery + chemotherapy + radiation	8.64%	6.41%	7.91%	7.81%	7.91%	7.26%	6.76%	NS
	Other/Unknown	17.03%	20.91%	23.10%	21.67%	21.86%	23.42%	26.97%	< 0.001 (+)
**Larynx**	Surgery	14.37%	11.96%	11.52%	13.35%	13.08%	13.24%	13.41%	NS
	Radiation	30.56%	29.44%	29.12%	26.23%	27.49%	27.44%	25.74%	< 0.001 (-)
	Chemotherapy + radiation	12.23%	18.15%	21.30%	22.92%	22.57%	22.61%	24.27%	< 0.001 (+)
	Surgery + radiation	24.63%	21.28%	15.74%	14.88%	14.54%	13.81%	12.30%	< 0.001 (-)
	Surgery + chemotherapy + radiation	4.78%	4.97%	7.08%	7.29%	6.55%	6.48%	6.57%	< 0.05 (+)
	Other/Unknown	13.44%	14.21%	15.25%	15.34%	15.78%	16.42%	17.71%	< 0.001 (+)
**Nasopharynx**	Surgery	3.21%	2.38%	2.48%	1.41%	1.61%	2.15%	1.76%	< 0.01 (-)
	Radiation	14.42%	11.15%	8.46%	8.63%	9.35%	7.43%	7.38%	< 0.001 (-)
	Chemotherapy + radiation	49.21%	57.99%	59.46%	61.63%	61.61%	62.31%	62.57%	NS
	Surgery + radiation	4.44%	3.80%	1.87%	2.94%	2.02%	1.97%	0.76%	< 0.01 (-)
	Surgery + chemotherapy + radiation	13.88%	7.15%	7.13%	7.83%	6.19%	4.76%	4.28%	NS
	Other/Unknown	14.83%	17.53%	20.60%	17.56%	19.23%	21.37%	23.26%	< 0.001 (+)
**Oral cavity**	Surgery	43.43%	45.39%	43.93%	43.02%	42.90%	42.21%	40.51%	< 0.01 (-)
	Radiation	9.92%	6.87%	5.19%	4.56%	3.57%	3.58%	4.16%	< 0.001 (-)
	Chemotherapy + radiation	4.42%	7.01%	7.31%	8.19%	6.10%	6.60%	3.91%	NS
	Surgery + radiation	26.55%	20.61%	17.32%	17.38%	19.48%	17.65%	19.64%	< 0.05 (-)
	Surgery + chemotherapy + radiation	4.42%	6.66%	11.99%	11.61%	14.35%	13.99%	13.45%	< 0.001 (+)
	Other/Unknown	11.25%	13.46%	14.25%	15.23%	13.61%	15.98%	18.34%	< 0.001 (+)
**Oropharynx**	Surgery	10.27%	7.20%	8.57%	6.94%	7.39%	7.69%	6.55%	NS
	Radiation	19.68%	15.70%	10.55%	9.79%	11.52%	12.45%	11.87%	< 0.01 (-)
	Chemotherapy + radiation	29.23%	41.68%	41.59%	46.01%	43.45%	41.63%	42.78%	< 0.001 (+)
	Surgery + radiation	14.33%	6.85%	5.02%	4.09%	4.92%	6.53%	6.17%	< 0.001 (-)
	Surgery + chemotherapy + radiation	7.24%	6.49%	9.09%	7.32%	9.37%	6.80%	7.15%	NS
	Other/Unknown	19.25%	22.08%	25.18%	25.86%	23.35%	24.90%	25.48%	< 0.01 (+)
**Other mouth**	Surgery	38.33%	37.31%	38.05%	37.83%	36.06%	35.75%	35.49%	< 0.001 (-)
	Radiation	12.72%	11.00%	8.01%	6.97%	6.98%	7.33%	5.80%	< 0.001 (-)
	Chemotherapy + radiation	7.57%	10.12%	12.34%	12.43%	11.89%	10.40%	7.41%	NS
	Surgery + radiation	19.90%	18.88%	14.58%	13.33%	14.59%	15.83%	18.67%	NS
	Surgery + chemotherapy + Radiation	3.96%	4.09%	7.52%	8.53%	9.04%	9.40%	10.36%	< 0.001 (+)
	Other/Unknown	17.52%	18.60%	19.49%	20.90%	21.43%	21.29%	22.26%	< 0.001 (+)
**Salivary gland**	Surgery	37.15%	34.47%	32.99%	32.75%	32.58%	33.16%	32.59%	< 0.05 (-)
	Radiation	4.03%	3.92%	3.36%	3.81%	4.09%	4.35%	4.30%	NS
	Chemotherapy + radiation	1.74%	2.35%	3.10%	3.15%	3.75%	3.27%	2.67%	< 0.05 (+)
	Surgery + radiation	43.00%	41.45%	39.84%	36.78%	33.94%	33.76%	32.49%	< 0.001 (-)
	Surgery + chemotherapy + radiation	4.90%	5.77%	8.29%	8.62%	8.96%	9.16%	8.65%	< 0.001 (+)
	Other/Unknown	9.17%	12.04%	12.42%	14.89%	16.68%	16.30%	19.30%	< 0.001 (+)
**Tongue**	Surgery	31.69%	29.79%	29.81%	29.10%	27.90%	29.02%	26.43%	NS
	Radiation	10.29%	8.96%	5.72%	4.98%	6.11%	7.03%	6.23%	< 0.01 (-)
	Chemotherapy + radiation	15.78%	26.57%	31.56%	32.37%	31.22%	29.63%	30.55%	< 0.01 (+)
	Surgery + radiation	20.50%	12.20%	9.35%	8.96%	10.00%	11.23%	12.00%	< 0.05 (-)
	Surgery + chemotherapy + radiation	9.10%	8.59%	10.36%	10.72%	11.07%	9.69%	10.29%	< 0.05 (+)
	Other/Unknown	12.65%	13.87%	13.21%	13.87%	13.71%	13.40%	14.50%	< 0.01 (+)

**Abbreviations:** HNC, head and neck cancer; NS, not significant.

**Fig. 3:**
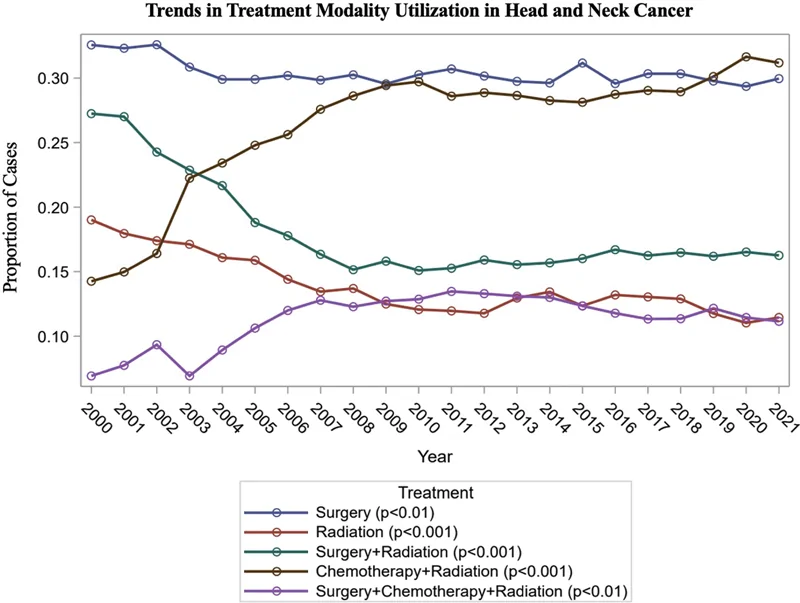
Trends in the proportion of all cases of head and neck cancer treated with each modality by year of diagnosis from 2000–2021. The *other/unknown* treatment category is not displayed.

### Five-Year Cancer-Specific Survival

Five-year cancer-specific survival trends varied by tumor site ( Fig. 4 , Table 2 ), with Fig. 4 demonstrating gradual improvements in survival for several subsites over time. Significant improvements in survival were observed for tongue, hypopharyngeal, oropharyngeal, and oral cavity cancers over the study period. Survival gains were modest in magnitude but consistent across time. No significant changes in 5-year survival were observed for nasopharyngeal or lip cancers. Laryngeal cancer and salivary gland malignancies demonstrated small but statistically significant improvements in survival.

**Fig. 4:**
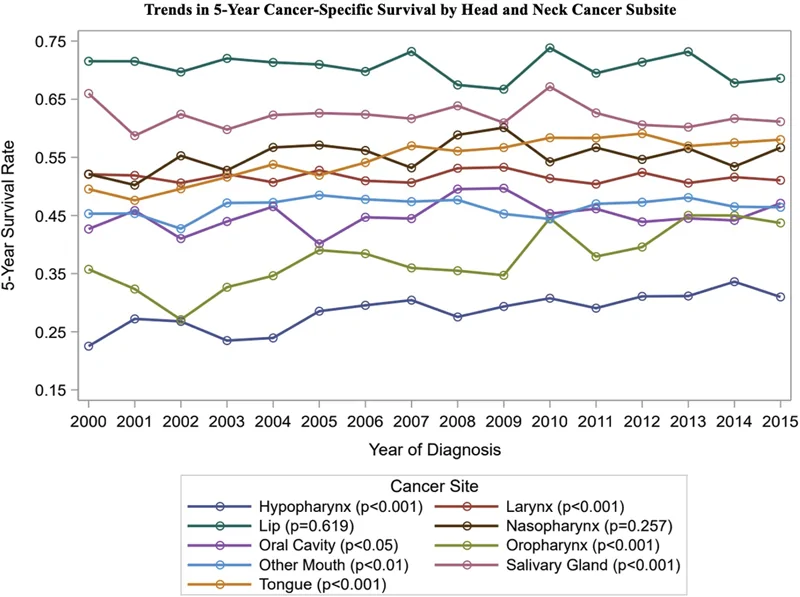
Five-year cancer-specific survival by tumor site according to year of diagnosis for cases diagnosed between 2000 and 2015.

### Missing Data

Stage information was unavailable for 6.1% of cases overall ( Table 1 ). The proportion of unstaged cases increased over time for lip, tongue, oropharyngeal, and other mouth/pharyngeal cancers, while remaining stable for other subsites ( Table 3 ). Treatment modality was classified as *other* in 13.8% of THE cases, reflecting chemotherapy-only treatment or missing data, and it increased significantly across all tumor sites ( Table 4 ).

### Summary of Major Findings

Across the study period, HNC demonstrated substantial site-specific heterogeneity in tumor distribution, stage at presentation, treatment approach, and survival outcomes. Tongue and oropharyngeal cancers increased in proportional representation and demonstrated improvements in survival, while the proportion of laryngeal and hypopharyngeal declined and showed greater reliance on chemoradiation. Overall, treatment strategies shifted away from radiation alone and surgery-radiation combinations toward increased use of chemoradiation and multimodal therapy.

## Discussion

This analysis of SEER data demonstrates substantial shifts in tumor-site distribution, stage at presentation, and treatment strategies from 2000 to 2021, with survival trends evaluated for cases diagnosed between 2000 and 2015.

### Survival Trends by Subsite

The most pronounced increases in survival were amongst oropharyngeal, tongue, and hypopharyngeal cancers. With regard to oropharyngeal and tongue cancers, possible contributing factors include improved diagnostic imaging, advanced surgical approaches such as transoral robotic surgery (TORS), and positron-emission tomography (PET) surveillance protocols to monitor for progression or recurrence. 7 8 Additionally, the rise in HPV-associated oropharyngeal cancer, which has a favorable prognosis compared to HPV-negative disease, could play a role in these findings. 9 10 In contrast, survival improvements in hypopharyngeal cancer are more likely associated with advancements in chemoradiation and multidisciplinary management. 11 12 Comparable trends have been reported in prior SEER analyses and institutional series, 12 supporting the durability of this shift.

Survival for nasopharyngeal cancers did not significantly improve despite measurable changes in treatment patterns. This may reflect later-stage presentation and more aggressive disease biology, potentially related to the absence of effective screening strategies. In addition, while treatment patterns for these subsites have evolved over time, findings from the current literature on the topic remain divided on whether these changes have translated into significant improvements in survival. While Sun et al. 13 reported improved survival in nasopharyngeal cancer, our findings were more consistent with those by Wu et al., 14 who demonstrated that combination therapies may improve survival in early-stage disease, while late-stage presentation remains associated with poor prognosis regardless of treatment. Finally, no significant survival gains were observed in lip cancers. This may reflect increased awareness of sun exposure, earlier identification due to anatomical visibility, or a plateau in treatment effectiveness.

### Tumor-Distribution Patterns and Etiologic Considerations

Changes in the proportional distribution of HNC subsites over time may reflect evolving etiologic patterns within the population. The proportion of tongue cancers increased steadily over the study period, which may be associated with shifts in underlying risk factors, including HPV-associated disease, as well as residual effects of prior tobacco and alcohol exposure and differences in detection and classification. 10 In contrast, the relative proportion of laryngeal and hypopharyngeal cancers declined, a pattern that is consistent with previously-reported reductions in tobacco and alcohol use at the population level and the impact of public health interventions. 15 Although salivary gland and nasopharyngeal cancers demonstrated small changes in proportional representation over time, these differences were modest and likely reflect their rarity and relatively-stable risk-factor profiles.

While the current analysis does not report population-based incidence rates, prior epidemiologic studies have demonstrated increasing incidence of HPV-associated oropharyngeal cancers, a trend that may contribute to the observed redistribution of tumor subsites 9 10 16 . However, the interpretation of tongue cancer trends in the present study was limited by the inability to distinguish oral tongue tumors from those of the base of the tongue within SEER. Although the rise in tongue-cancer proportions coincides with increasing rates of HPV-associated disease reported in the literature, these findings likely reflect a heterogeneous group of tumors with differing etiologies, and they should not be interpreted as directly attributable to HPV. 16 The absence of HPV status within SEER further limits differentiation between HPV-associated and non–HPV-associated disease, and consideration of prior literature should therefore be viewed as contextual rather than explanatory.

### Treatment Trends and Evolving Standards of Care

The proportion of cases using chemoradiation increased, while those reporting the use of radiation alone and surgery-radiation declined. These changes align with key organ-preservation trials such as those by Forastiere et al. 17 and the Department of Veterans Affairs Laryngeal Cancer Study Group, 18 which demonstrated that chemoradiation provides improved outcomes compared to radiation alone while preserving organ function. At the same time, trimodal therapy increased for select subsites, suggesting a shift toward more intensive multimodality treatment in patients with advanced or high-risk disease. In contrast, the decline in surgery-radiation may reflect de-escalation strategies for cases detected at an earlier stage, which carry a more favorable prognosis and do not always require aggressive multimodality treatment. In these cases, the goal is effective tumor control while preserving function, unlike treatment strategies for more aggressive subtypes. Surgery still remains central for early-stage disease—particularly in oral cavity cancers—in which primary resection often yields superior local control. Finally, treatment evolution has been shaped by the rise of multidisciplinary cancer care. Tumor-board conferences have fostered closer collaboration among specialties and support more coordinated, evidence-based decision-making.

### Clinical Stage Migration and Implications

The observed shift toward a higher proportion of regional and distant disease may reflect improved surveillance leading to better detection of local tumors and metastases, evolving disease biology, and potential diagnostic delays. 19 20 This migration has significant treatment implications: advanced-stage presentation often requires multimodality therapy and is associated with poorer outcomes, whereas earlier detection allows for less intensive treatment. 21 22 These trends may also contribute to increased chemotherapy use for curative and palliative intents in advanced disease. Monitoring staging patterns is essential to guide public-awareness efforts and further improve survival. Importantly, the observed increase in regional and distant-stage diagnoses should be interpreted alongside rising proportions of unstaged cases. This pattern may reflect changes in documentation practices rather than true biological shifts.

### Implications for Health Systems and Policy

Improvements in survival for tongue and oral-cavity cancers may reflect changes in risk-factor exposure, earlier detection, and evolving treatment strategies. These findings reinforce the importance of site-specific treatment strategies and early-stage detection, particularly for subsites demonstrating increasing advanced-stage presentation. The observed shift toward chemoradiation further supports the need for coordinated multidisciplinary care, especially in complex or advanced disease. Persistent variation in survival across subsites highlights potential disparities in stage at diagnosis and access to comprehensive treatment. Leveraging SEER data can help identify gaps in care and resource allocation, guiding targeted interventions and health-policy efforts to improve equity in HNC management.

### Limitations and Future Directions

The current study has several limitations inherent to the use of SEER data. First, tumor-site classification does not strictly align with American Joint Committee on Cancer (AJCC) definitions, resulting in aggregation of anatomically-distinct subsites. 23 Additionally, broader categories such as *other mouth* further group heterogeneous anatomical regions that would ideally be classified within oral-cavity or oropharyngeal subsites. These limitations are important, as cancers originating from the base of the tongue are more strongly associated with HPV, whereas cancers of the oral tongue and other oral-cavity subsites are typically HPV-negative and demonstrate different risk profiles and clinical behavior. Consequently, observed trends in “tongue” and other aggregated oral-cavity categories likely reflect heterogeneous populations and should be interpreted with caution.

Second, SEER lacks detailed clinical variables, including HPV and Epstein-Barr virus (EBV) status, systemic therapy regimens, and functional or quality-of-life outcomes. Third, the current study reports proportional distributions within the HNC cohort rather than population-based incidence rates, and survival analyses do not account for comorbidities or baseline functional status which are not available in SEER.

Finally, treatment classification is limited by the *other* category, which includes chemotherapy-only cases and missing or incomplete treatment data. The proportion of this category increased over time, introducing potential misclassification that may influence observed treatment trends. Consequently, changes in the use of specific modalities may be partially attributable to incomplete reporting rather than true shifts in clinical practice. Despite these limitations, SEER remains one of the most comprehensive sources for population-level cancer data in the US. Future studies incorporating more granular clinical data, improved completeness of treatment reporting, age-standardized incidence rates, subgroup analyses by stage and treatment, and interaction modeling are needed to further clarify these trends and their clinical implications.

## Conclusion

Over the past 2 decades, survival for several HNC subsites has improved, alongside observed shifts in tumor distribution, stage at presentation, and treatment patterns. Greater use of chemoradiation and trimodal therapy likely reflects evolving treatment strategies tailored to tumor site and disease severity. Despite these advances, survival patterns were not consistent across all subsites, and the higher proportion of advanced-stage disease in several subsites highlights ongoing challenges related to early detection and access to care. Variation in outcomes underscores the importance of site-specific treatment approaches and coordinated multidisciplinary management. Data from the SEER remain a valuable resource to characterize national patterns and identify opportunities to improve cancer-care delivery, inform public-health efforts, and guide future research.
